# Cadmium Modulates Biofilm Formation by *Staphylococcus epidermidis*

**DOI:** 10.3390/ijerph120302878

**Published:** 2015-03-04

**Authors:** Xueqing Wu, Regiane R. Santos, Johanna Fink-Gremmels

**Affiliations:** Institute for Risk Assessment Sciences, Division Veterinary Pharmacology, Pharmacotherapy and Toxicology, Faculty of Veterinary Medicine, Utrecht University, P.O. Box 80152, 3584 CM Utrecht, The Netherlands; E-Mails: x.wu@outlook.com (X.W.); R.Rodriguesdossantos@pq.cnpq.br (R.R.S.)

**Keywords:** ATCC35984, *Staphylococcus epidermidis*, cadmium, biofilm, polysaccharide intercellular adhesion, accumulation-associated protein

## Abstract

The aim of the study was to evaluate the effect of cadmium exposure on *Staphylococcus epidermidis* (ATCC 35984) biofilm formation. Bacteria were cultured in the absence or presence of different concentrations (0–50 µM) of cadmium. Biofilm formation and bacterial viability were assessed. Quantitative Real Time-PCR (qRT-PCR) was used to determine the mRNA expression of molecular markers of *S. epidermidis* biofilm formation and dispersion. *S. epidermidis* biofilm formation was stimulated (*p* < 0.001) by 1.56 and 3.13 µM cadmium. Confocal laser scanning microscopy (CLSM) analysis confirmed an increase in biofilm thickness (23 and 22 µm, *versus* 17.8 µm in the controls) after exposure to 1.56 or 3.13 µM cadmium, respectively. qRT-PCR was performed showing the up-regulation of *atlE, embp, aap, icaA* and *icaB* after exposure to 3.13 µM cadmium. Taken together, these findings show that cadmium at low, sub-toxic concentrations acts as inducer of *S. epidermidis* biofilm formation.

## 1. Introduction

Heavy metal contamination of soil and water is a worldwide risk affecting food and feed safety [1,2]. Among various heavy metals, cadmium is considered to be the most toxic element, predominantly due to its long biological half-life resulting in accumulation in the body, particularly the kidneys [3]. Plants can take up cadmium from soil and hence cadmium is a regular contaminant of food and animal feeds [4]. The hazard of cadmium was well studied in past decades, and has been recently summarized in a broad exposure analysis by the World Health Organisation [5].

Sub-toxic concentrations of heavy metal have been described as potential inducers of biofilm formation [6]. This effect might be desirable, when microorganisms are used to decontaminate soil or water in industrial areas, but is undesirable in pathogenic bacteria, as biofilm formation confers resistance to most commonly used antibiotics and protects pathogens from host defence mechanisms [7,8]. One of the organisms known for its rapid biofilm formation is *Staphylococcus epidermidis*, which can be isolated from soil [9] and wastewater [10], but is also a common commensal organism on the human skin and a facultative pathogen [8].

Bacterial biofilm formation can be stimulated by diverse stress factors, such as drugs [11] or heavy metals from the environment. For example, Perrin *et al.* [6] reported that sub-inhibitory concentration of nickel promotes *Escherichia coli* K-12 biofilm formation. Schue *et al.* [12] showed that cadmium triggers *Rhizobium alamii* biofilm formation. The *S. epidermidis* strain ATCC 35984 contains putative cadmium resistance genes and it has been suggested that these are associated with its ability to readily form biofilms [13].

The aim of the current study was to assess the effect of cadmium on *S. epidermidis* viability and its ability to form biofilms. As an experimental model, two ATCC type strains of *S. epidermidis* were compared*.* The ATCC 12228 strain does not produce biofilms, as it lacks the *ica* operon, and was used to establish the MIC values for cadmium chloride. In contrast, ATCC 35984, forms biofilms [8]. Recent investigations by Mertens and Chebremedhin *et al.* [14] indicated that approximately 50% of all clinical isolates of *S. epidermidis* are genetically positive for this *ica* operon. To demonstrate the association of cadmium tolerance and the capacity of ATCC 35984 to form biofilms, a detailed analysis of the induction of biofilm formation, biofilm architecture and the expression of genes encoding key regulators of biofilm formation is presented.

## 2. Materials and Methods

Unless mentioned otherwise, chemicals used in the present study were purchased from Sigma-Aldrich (St. Louis, MO, USA).

### 2.1. Bacterial Strains and Culture Conditions

Two strains of *S. epidermidis* were purchased from the American Type Culture Collection (ATCC), (LGC Standards GmbH, Wesel, Germany). The strain ATCC 35984 (RP62a-corresponding to ST10) forms a thick biofilm on the bottom of a 96-well plate, affecting optical density (OD) measurement required for a quantitative MIC determination. Therefore, we used the non-biofilm-forming strain (ATCC 12228-corresponding to ST8) for the measurement of MIC and minimal bactericidal concentration (MBC). Both strains were maintained on tryptic soy broth (TSA) (Oxoid CM 129) slants at 4 °C. Before use, one colony of bacteria was cultured in 10 mL tryptic soy broth (TSB) + 0.25% glucose (TSB^+^) (pH 7.0) under aerobic conditions at 37 °C for 24 h.

### 2.2. Cadmium

A 0.2 M solution of cadmium (Cd^2+^) (cadmium chloride) was prepared and stored at 4 °C. Before use, the stock solution was diluted in TSB^+^ to a final concentration of 100 μM.

### 2.3. Bacterial Susceptibility to Cadmium

The minimum inhibition concentration (MIC) and minimum bactericidal concentration (MBC) were determined using the non-biofilm forming strain (ATCC 12228), following the Clinical and Laboratory Standards Institute standard broth micro-dilution method with slight modifications. Briefly, serial two-fold dilutions of 100 µM cadmium in TSB^+^ were prepared down to a final concentration of 0.78 µM cadmium, in a U-bottom 96-well plate (100 µL/well). To each well, 100 µL of 10^6^ CFU (colony-forming unit)/mL bacterial suspension was added, resulting in a final volume of 200 µL and final concentrations of cadmium of 0.39, 0.78, 1.56, 3.13, 6.25, 12.5, 25, and 50 µM. Wells with sterile TSB^+^ alone served as blanks. Plates were incubated at 37 °C for 24 h. Thereafter, 100 µL of the incubated suspension was transferred into a new sterilized flat bottom 96-well plate and its OD was measured at 655 nm wavelength using a microplate reader (model 3550, Bio-Rad, Hercules, CA, USA). MIC was defined as the lowest cadmium concentration that inhibited visible growth after 24 h of culture and resulted in an OD value similar to the blank (TSB^+^ only). After these 24 h incubation at 37 °C, aliquots of 10 µL from each well were spotted on to tryptic soy agar (TSA) plates containing no cadmium. The MBC was read as the lowest cadmium concentration with no growth after 24 h culture. All MIC/MBC experiments were carried out in triplicate with three independent repetitions.

### 2.4. Quantification of Biofilm Formation

Biofilm formation was assessed by the standard safranin colorimetric assay as described previously [15,16] using the strain ATCC 35984. In brief, 100 µL of bacterial suspension (10^6^ CFU/mL) was transferred into a U-bottom 96-well microtiter polystyrene plate (Costar, Corning, NY, USA). To this suspension, a solution of TSB^+^ containing cadmium at concentrations of 0.78, 1.56, 3.13, 6.25, 12.5, 25, 50, or 100 µM was added, resulting in final concentrations of cadmium are 0.39–50 µM in the tested samples. Wells with sterile TSB^+^ alone served as blanks. The plates were incubated on a microplate shaker (Titramax 100, Heidolph, Schwabach, Germany) at 600 rpm, 37 °C for 24 h. At the end of the culture period, the supernatants from all wells were discarded and the biofilms adhered to the bottom of the wells were incubated with 0.1 M HCl for 1 h at room temperature. Thereafter, HCl was replaced by safranin (0.1% in water) and the plates were incubated for 45 min at room temperature. Non-bound safranin was removed by rinsing the wells three times with de-ionized water, and thereafter plates were incubated with 125 µL 0.2 M NaOH per well at 57 °C for 1 h. At the end of incubation, 100 µL from the stained dissolved biofilm in each well was pipetted into a new flat-bottom 96-well microtiter polystyrene plate and its intensity was measured at a wavelength of 540 nm in a microplate reader. Minimum biofilm inhibitory concentration (MBIC) was defined as the lowest concentration that inhibited at least 90% biofilm formation. Each test was performed in quadruplicate with three independent repetitions.

### 2.5. Determination of Bacterial Viability in Suspension and in the Biofilm

To quantify the number of culturable (*i.e.*, actively growing) bacterial cells in suspension and in the biofilm after exposure to different concentrations of cadmium (0.39–50 µM), a plating method was conducted as described by Cabal *et al.* [17], with some modifications. Briefly, for counting the bacteria in suspension (planktonic bacteria), 200 µL of supernatant was separately taken from each well, without disturbing the biofilm, and subjected to 10-fold serial dilutions in TSB^+^. Subsequently 10 drops of 10 µL supernatant samples from each well were plated onto TSA and cultured for 24 h at 37 °C. After culture, drops that had 3–30 counts were chosen to calculate the number of viable bacteria in suspension by the formula: N (mean value of colony number from 10 drops) × 100 × 2 = CFU/well (200 µL) (Figure S1). For counting the bacteria in biofilm, plates with biofilm attached on the bottom were carefully washed three times with sterile saline solution to remove free cells before adding 200 µL TSB^+^. Biofilms from each well were scraped and then dispersed by using a 0.5 × 16 mm needle until no visible flocculent was observed in the suspension. Bacteria obtained from biofilms were diluted, plated, and the total number of viable culturable cells in the biofilm was calculated and expressed as CFU/well by following the same methods used for the bacteria in the suspension. Experiments were carried out in triplicate with three independent repetitions.

### 2.6. Bacterial Viability and Biofilm Thickness Determined by Confocal Laser Scanning Microscopy

Biofilms of ATCC 35984 were formed after culture at 37 °C for 24 h on coverslips inserted into tubes containing either control medium or medium supplemented with cadmium at a range of test concentrations (0.39–50 µM). After culture, coverslips carrying biofilms were incubated for 15 min in the dark at 37 °C with 1 nM SYTO^®^ green fluorescent nucleic acid dye and 6 nM propidium iodide from propidium/SYTO green viability staining kit (Life Technologies Europe BV, Bleiswijk, The Netherlands). Biofilms were washed three times in PBS and then examined using confocal laser scanning microscopy (CLSM) (Leica TCS SPE-II, Mannheim, Germany). Bacteria in biofilms were classified as viable if not stained positively by propidium (red). The excitation/emission wavelength for these dyes were 480/500 nm for SYTO^®^ green and 490/635 nm for propidium iodide. To estimate the percentages of dead bacteria, the program Image J 1.47 was used to count the propidium stained cells (given as cell area). Biofilms were analysed by a series of images in the z-axis, followed by digitized images in selected optical planes with a Leica CLSM (Leica TCS SPE-II, Mannheim, Germany). Automated on-line collection of confocal two-dimensional cross-sectional images was used to determine biofilm architecture from a three-dimensional reconstruction by Image J 1.47.

### 2.7. Quantitative RT-PCR

RNA was isolated from planktonic bacteria and those embedded in biofilm (the strain ATCC 35984 was used) using the Trizol reagent and FastPrep^®^ Disrupter (Thermo Savant, Qbiogene, Inc., Cedex France). Firstly, 1 mL of bacterial suspension or biofilm in TSB^+^ was centrifuged (15,000× g) for 10 min at 4 °C. The supernatant was removed and 1mL Trizol reagent was added and pipetted several times. The suspension was transferred to a Lysing Matrix E tube (MP Biomedicals Germany GmbH, Eschwege, Germany) and homogenized in the FastPrep^®^ FP120 Cell Distrputer (Thermo Savant, Qbiogene, Inc.) for two periods of 45 s at a speed of 6.5 m/s. Subsequently, the samples were centrifuged (15,000× g) for 5 min at 4 °C, and the supernatants were separately transferred to 1.5 mL Eppendorf tubes and subjected to the phenol-chloroform RNA extraction protocol. The concentration and purity of total RNA was spectrophotometrically assessed using a NanoDrop 1000^TM^ (Thermo Scientific, Waltham, MA, USA), and 1 µg of extracted total RNA from each sample was reverse transcribed with the iScript^TM^ cDNA Synthesis kit (Bio-Rad). The obtained cDNA was diluted to a final concentration of 30 ng/mL. Primers (Table 1) complementary to *S. epidermidis* were designed according to literature, and were commercially produced (Eurogentec, Maastricht, The Netherlands). The primers used were selected based on specificity and efficiency by qPCR analysis of a dilution series of pooled cDNA at a temperature gradient (55–65 °C) for primer-annealing and subsequent melting curve analysis. The reaction mixture for qPCR contained 10 μL of diluted cDNA, 12.5 μL iQSYBR Green Supermix (Bio-Rad), forward and reverse primers (final concentration of 0.4 pmol/μL for each primer) and sterile water according to the manufacturer’s instructions. qPCR was performed using the MyiQ single-colour real-time PCR detection system (Bio-Rad) and MyiQ System Software Version 1.0.410 (Bio-Rad). The relative mRNA expression was calculated from the comparison with the expression levels of two reference genes, heat shock protein 60(*hsp60*) and triosephosphate isomerase (*tpi*) (Table 1).

**Table 1:** Primer sequences used in this study.

Gene	GenBank	Primer	Sequence	Product Size (pb)	References
*hsp60*	AF029245	Forward	5’ GTTTTAGCACAATCAATGATTCAG 3’	491	[18]
		Reverse	5’ GCATCGCCTTCTACTTCATCC 3’		
*tpi*	AF269838	Forward	5’ CATCTGATAAACCTTCGACAGCTTT 3’	128	[19]
		Reverse	5’ TGCTATCTTCAATCACGGTATGACA 3’		
*aap*	AJ249487	Forward	5’ ATACAACTGGTGCAGATGGTTG 3’	400	[20]
		Reverse	5’ GTAGCCGTCCAAGTTTTACCAG 3’		
*agrB*	AF012132	Forward	5’ TTCGTTTAGGGATGCAGGTA 3’	141	[21]
		Reverse	5’ ATGGCACACGTACAGAGGAT 3’		
*atlE*	U71377	Forward	5’ TGTCCTGCTTTCACGTATGA 3’	139	[21]
		Reverse	5’ AGAAACCTTAACCACGTAAA 3’		
*embP*	AY101364.1	Forward	5’AGCGGTACAAATGTCAATATC 3’	455	[22]
		Reverse	5’AGAAGTGCTCTAGCATCATCC 3’		
*icaA*	U43366	Forward	5’ AACAAGTTGAAGGCATCTCC 3’	166	[23]
		Reverse	5’ GATGCTTGTTTGATTCCCT 3’		
*icaB*	U43366	Forward	5’ AATGGCTTAAAGCACACGAC 3’	144	[21]
		Reverse	5’ TTTGTCCTTTCCGTAACAGT 3’		
*sarA*	NC002976.3	Forward	5’ TGGTCACTTATGCTGACAGATT 3’	313	[24]
		Reverse	5’ TTTGCTTCTGTGATACGGTG 3’		
*sepA*	NC002976.3	Forward	5’ CGCACCAGACAACGCTGTA 3’	170	[25]
		Reverse	5’ TCAATCGCACATGTAAATAACTTCC 3’		
*rsbU*	NC002976	Forward	5’ TCTCTTCATACAGTCCAT 3’	172	[26]
		Reverse	5’ ATAGGTTCAGGTATTCCA 3’		

### 2.8. Statistical Analysis

Data were evaluated with one-way analysis of variance (ANOVA) using Graph Pad Prism version 6.04 for windows (Graph Pad Software, San Diego, CA, USA).

## 3. Results

### 3.1. Bacteriostatic and Bactericidal Effects of Cadmium on S. epidermidis

**Figure 1:**
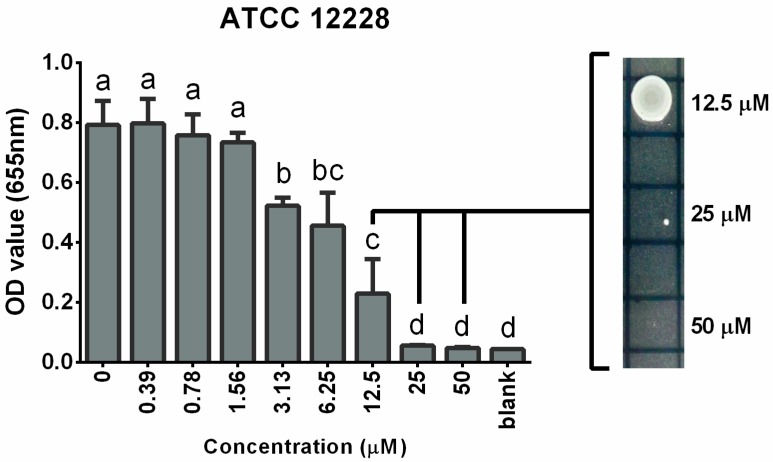
Mean (±SEM) OD values of *S. epidermidis* (ATCC 12228) after exposure to cadmium (0.39–50 µM); measured at 655 nm wavelength. Different letters (a–d) indicate significant difference between compared groups. 12.5, 25, and 50 µM cadmium treatment groups were chosen for drop plating (right side image).

MIC and MBC of cadmium were determined. The MIC of cadmium on *S. epidermidis* (ATCC 12228) was determined as 25 µM (Figure 1), as there was no difference between the OD values obtained after bacterial exposure to 25 µM cadmium and the negative control. Thereafter, by plating the cadmium-exposed (12.5, 25, and 50 µM) bacteria, we found that the MBC of cadmium on *S. epidermidis* (ATCC 12228) was 50 µM, as no bacterial colonies were present at this concentration.

### 3.2. Cadmium Increases S. epidermidis Biomass

**Figure 2:**
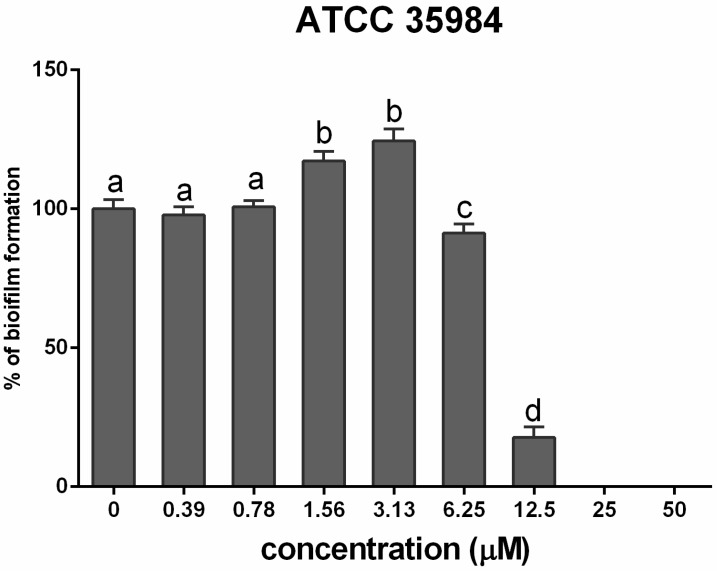
Mean (±SEM) percentage of biofilm formation by *S. epidermidis* (ATCC 35984) after exposure to different concentrations of cadmium. Different letters (a–d) indicate significant differences between compared treatment groups. MBIC was defined as the lowest concentration that inhibited at least 90% biofilm formation.

*S. epidermidis* biofilm formation and inhibition were quantified. *S. epidermidis* (ATCC 35984) biofilm formation was increased (*p* < 0.001) by cadmium at concentrations of 1.56 and 3.13 µM, but biofilm formation was inhibited at a cadmium concentration of 6.25 µM (Figure 2).The MBIC of cadmium on *S. epidermidis* (ATCC 35984) biofilm was 25 µM, at which concentration biofilm formation was inhibited by 90%.

### 3.3. Viable Counts in Suspension and in the Biofilm

**Figure 3:**
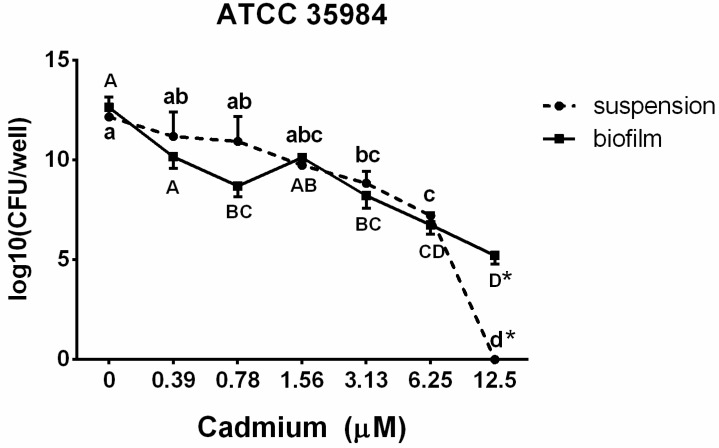
The bacterial population *S. epidermidis* (ATCC 35984) in suspension and biofilm after exposure to different concentrations (0.39–12.5 µM) of cadmium. Different letters indicate significant differences between cadmium concentrations in each suspension (lower case letters: a–d) or biofilm (upper case letters: A–D). * indicates significant difference between biofilm and suspension in same cadmium treatment group.

To evaluate the viability of the bacterial population in suspension and biofilm after exposure to cadmium, growth of the biofilm-positive strain *S. epidermidis* (ATCC 35984) was measured using the drop plate method. The results (Figure 3) showed a cadmium concentration-dependent decrease of bacterial counts in both the suspension and the biofilm. Bacterial counts in suspension were decreased (*p* < 0.05) after exposure to 3.13 µM cadmium and no culturable counts were observed after exposure to 12.5 µM cadmium. When enclosed in the biofilm, bacterial counts were diminished (*p* < 0.05) immediately after exposure to 0.78 µM cadmium. However, in contrast to bacterial suspension, 10^5^ CFU/well of bacterial counts in the biofilm were culturable even after exposure to 12.5 µM cadmium.

### 3.4. Bacterial Viability and Biofilm Architecture

**Figure 4:**
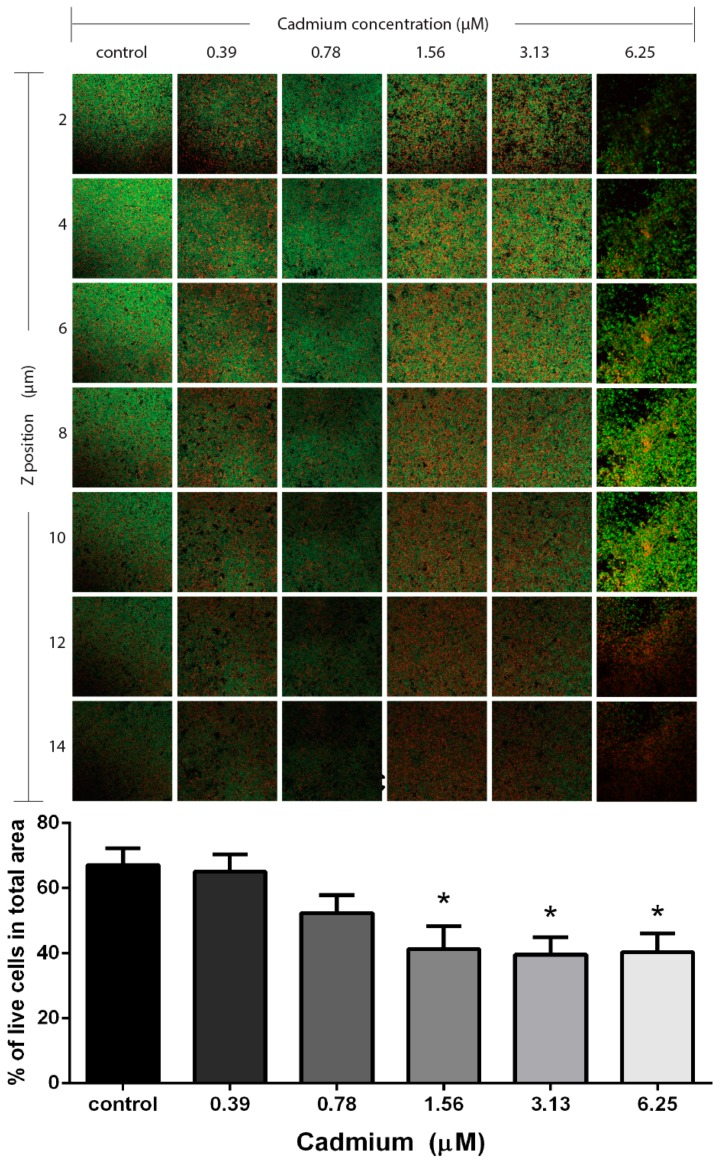
Bacterial viability in *S. epidermidis* (ATCC 35984) biofilms measured by confocal scanning laser microscopy. Non-viable bacteria were stained red, and total bacteria were stained green by using the propidium/SYTO green viability staining kit. Bacteria not stained in red were thus considered viable. Z-position images of controls and different cadmium concentrations (0.39–6.25 µM) were acquired in every 2 µm section. The graphic under the images shows the percentage of viable bacteria determined from all biofilm layers by image J 1.47.

Figure 4 illustrates our findings when fluorescent markers (propidium/SYTO green viability staining kit) were used to quantify the percentage of viable bacteria (ATCC 35984) encapsulated in matrix biofilms. Exposure to cadmium resulted in a decrease in viable bacteria after exposure to 1.56 (41.4 ± 7.0%) or 3.13 µM (39.6 ± 5.3%) cadmium, whereas the viability in control biofilms exceeded 60% (67.1 ± 5.2%). Similar results had been obtained with viability counts as presented in Figure 3. Noteworthy, non-viable bacteria were mostly found in the outer layers of the biofilm.

**Figure 5:**
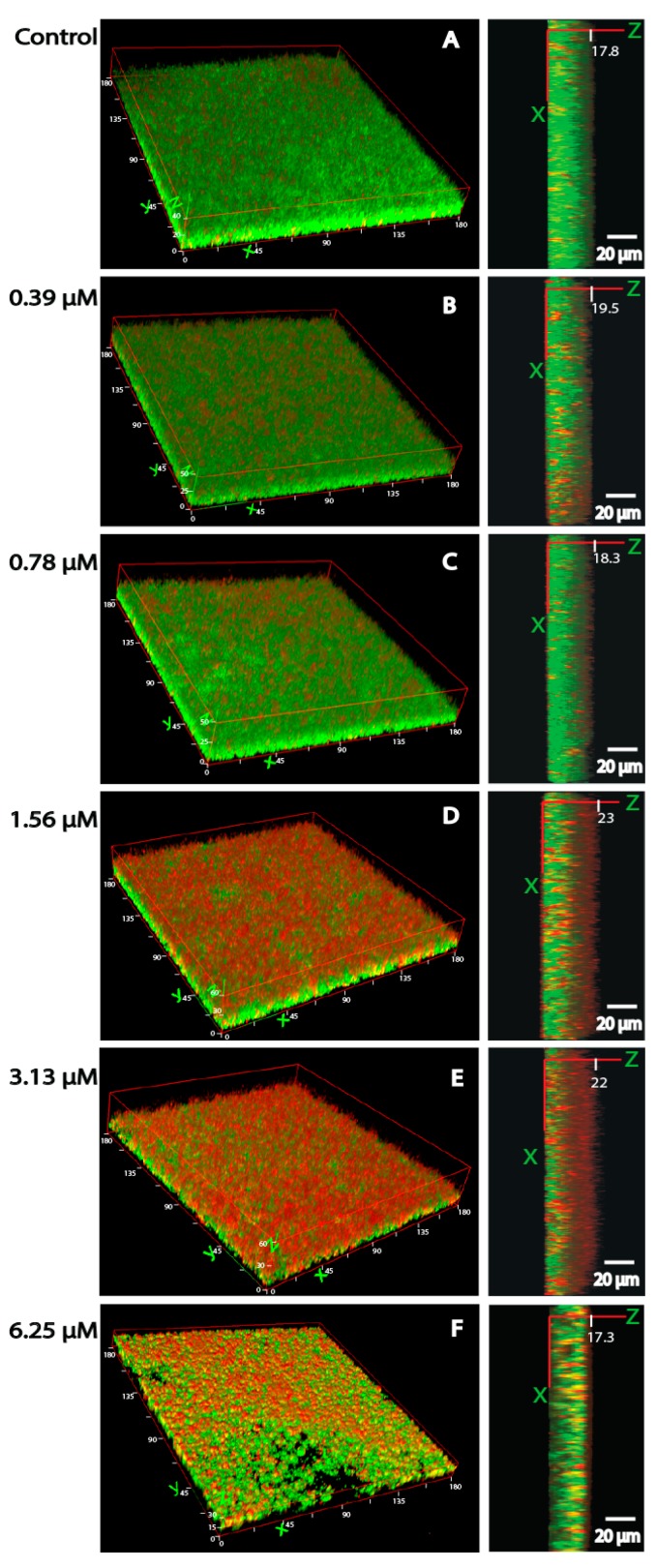
The thickness (right panel) and 3D (left panel) pictures of *S. epidermidis* (ATCC35984) measured by CLSM. Non-exposed (control) (**A**) or exposed to different concentrations (0.39–6.25 µM) (**B**–**F**) of cadmium. The thicknesses of biofilms from control to 6.25 µM treatment group were 17.8, 19.5, 18.3, 23, 22, and 17.3 µm, respectively. Non-viable bacteria were stained red, and viable bacteria were stained green by using the propidium/SYTO green viability staining kit.

When a 3D reconstruction was performed (Figure 5), it was observed that control samples presented a compact and dense biofilm with a thickness of 17.8 µm. Biofilms became thicker with increasing cadmium concentrations, reaching the highest values after exposure to 1.56 µM (23 µm) and 3.13 µM (22 µm) cadmium. However, when the cadmium concentration was increased to 6.25 µM, biofilm thickness (17.3 µm) decreased significantly with a notable alteration in the biofilm architecture.

### 3.5. Gene Expression in Planktonic Cells and Mature Biofilms

**Figure 6:**
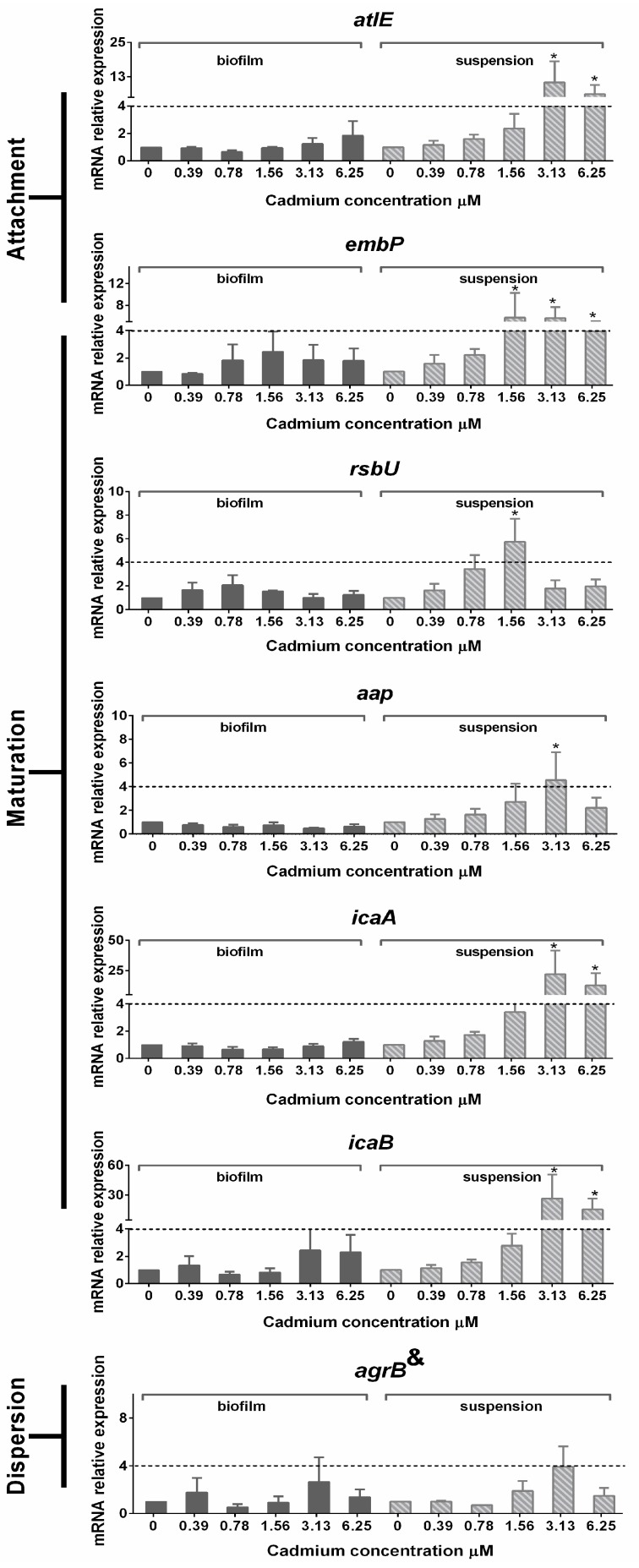
Mean (±SEM) relative expression of mRNA encoding genes involved in biofilm formation and quorum sensing. * indicate significant relative down- or up-regulation of genes when compared with untreated controls. ^&^ indicate *agrB* is considered to be involved in bacterial attachment and biofilm dispersion.

The relative mRNA expression levels of *sarA* and *sepA* in suspension and biofilm were unaltered compared to the untreated controls after treatment of *S. epidermidis* with different concentrations of cadmium (0.39–6.25 µM) (data not shown). The relative mRNA expression levels of *atlE*, *embp*, *rsbU*, *icaA*, *icaB,* and *agrB* in planktonic bacteria and biofilm-embedded bacteria measured 24 h after culture are depicted in Figure 6. *AltE*, *embp*, *icaA* and *icaB* were up-regulated (≥4 fold) in suspension when bacteria were treated with 3.13 and 6.25 µM cadmium, but none of them were altered in biofilm-embedded bacteria. In planktonic bacteria, *RsbU* mRNA expression was up-regulated when exposed to 0.78 and 1.56 µM cadmium. The relative mRNA expression of *aap* was up-regulated in suspension when bacteria were treated with 3.13 µM cadmium.

## 4. Discussion

As yet, most studies involving the effect of heavy metals on bacteria have focused on the bacterial biosorption ability, including binding to biofilms. Only occasionally studies emphasize the effect of heavy metals on bacterial biofilm formation in commensal and potential pathogenic bacteria, such as clinical isolates of *S. epidermidis*, although biofilm formation is one of the most common causes of antimicrobial resistance [14,27]. Investigations with 300 clinical isolates of blood cultures and indwelling devices indicated that are indeed 32% of these clinical isolates are biofilm producers (measured on Congo Red Agar). However, as also 3 *icaA/D*-negative strains were found to produce phenotypically biofilms, the authors suggested that other factors such as sub-inhibitory concentrations of antibiotics and stress factors might have a significant role in biofilm formation.

Perrin *et al.* [6] reported that *E. coli* (K-12) exposure to 100 µM nickel promoted biofilm formation, but data on the effect of cadmium are lacking. It is therefore of interest that we could show that cadmium indeed stimulated *S. epidermidis* (ATCC 35984) biofilm formation at concentrations of 1.56 and 3.13 µM, whereas pilot experiments with other metals, such as lead, mercury, nickel and manganese did not indicate that these metals affected biofilm formation in *S. epidermidis* (Figure S2).

At higher concentrations, cadmium exerted an antibacterial effect. This inhibition was observed at a concentration range from 6.25 to 50 µM. Shaivastave *et al.* [28] reported that the MIC of cadmium on *S. aureus* isolates from industry wastewater was 450 µg/mL (~2.45 mM), which is much higher than the concentration that was effective against *S. epidermidis* (ATCC 12228) in our *in vitro* study. AbubAkr *et al.* [29] showed *in vitro* that the *S. sciuri* (18 strains) MIC values range between 10–300 μg/mL (54–1633 μM). These values are also much higher than the MIC values reported here indicating the specific sensitivity of *S. epidermidis*. The MBIC, MIC and MBC concentrations were found to be in the same range, amounting for *S. epidermidis* (ATCC 35984 and ATCC 12228) to 25, 25 and 50 µM, respectively. These minor differences between a biofilm producer and a non-biofilm producing strain suggest that sensitivity and resistance to cadmium are not only mediated by the ability of *S. epidermidis* (ATCC 35984) to form readily biofilms. This is also suggested by Gill *et al.* [13], who identified a plasmid denoted vSe1. This plasmid has prophage integrase genes in the biofilm forming strain RP62A (ATCC 35984), but direct evidence for its involvement in cadmium tolerance was not shown.

Of medical and also eco-toxicological importance is the biofilm stimulation that was observed after exposure to very low concentrations of cadmium, such as 1.56 or 3.13 µM. These results were confirmed by our CLSM analysis, which indicated the increase in biofilm thickness after exposure to cadmium at concentrations of 1.56 µM (22 µm) and 3.13 µM (23 µm) together with a loss of bacterial viability at the same concentrations. This loss of viability was mainly observed in the outer layer of the biofilms. Similar results were obtained with *Pseudomonas aeruginosa* exposed to copper, where the highest percentage of dead biomass was found in the outer layer of the biofilm [30]. It was suggested that this phenomenon is related to the fact that bacteria in the outer layers of a biofilm are exposed to the highest concentrations of a toxic agent, whereas bacteria in the central parts of a biofilm are protected by the biofilm matrix.

For a better understanding of the mechanism involved in biofilm formation and maturation, the relative mRNA expression during *S. epidermidis* biofilm formation was determined. No significant changes were noted in bacteria embedded in a biofilm. In contrast, concentration-dependent changes following cadmium exposure were observed in planktonic persister cells. For example, in persisters, *rsbU*, a stress marker [31], was already up-regulated at low concentrations (0.78 and 1.56 µM Cd), and subsequent up-regulation of biofilm related genes was expected. Genes related to attachment such as *atlE* and *embp* were up-regulated, comparable to previous findings where this upregulation was associated with the attachment to a polystyrene device [20] and the intercellular adhesion during bacterial attachment [32]. Concomitantly, *icaA* and *icaB* were up-regulated not only as a stimulus to intercellular adhesion, but also because they regulate PIA synthesis, a main agglutination agent in the biofilm formation of *S. epidermidis* [8,33]. Transcription of *aap* was increased in planktonic persister bacteria during exposure to 3.13 µM cadmium. This gene supports bacterial accumulation [33], and is one of the most important proteins involved in *S. epidermidis* biofilm formation [34]. *agrB* is a biomarker of *agr* quorum-sensing system [35]. This gene was slightly (non-significant) up-regulated after exposure to 3.13 µM cadmium in both planktonic and biofilm bacteria, indicating that cadmium did not affect the quorum-sensing system in the present study. The gene regulation in persisters may reflect the stimulatory effect of cadmium on *S. epidermidis*. This is in line with the hypothesis that as a commensal opportunistic pathogen, *S. epidermidis* can adapt to various environmental stress conditions. A typical example is the metal inducible TCS (two component system) allowing an adaptive response of bacteria to changing environmental conditions as it regulates the expression of various genes determining virulence factors and biofilm formation [36]. In the present study, cadmium was found to stimulate *S. epidermidis* biofilm formation by positively influencing bacterial attachment and biofilm maturation, and this may indeed be related to the two-component system (TCS).

As mentioned above, gene expression within the biofilm was not affected by cadmium. This phenomenon may be explained by the fact that bacterial cells in a biofilm are in dormant state. Hu *et al.* [34] also showed that *aap* transcription in planktonic *S. epidermidis* was increased, while it was decreased in biofilm-embedded bacteria. Pintens *et al.* [37] demonstrated that in the initial 4–7 h during biofilm formation, *aap* expression was transiently higher in sessile than in planktonic bacteria, after which it progressively declined in sessile bacteria. Most changes in bacterial biofilm gene expression were observed within the first 8 h of culture in *S. epidermidis* biofilm-embedded bacteria [36]. As in the current experiments gene expression was measured only after a 24 h culture period, *i.e.*, at a time point when the biofilm was already mature, such as stable gene expression could be expected. Comparably, it has been reported that gene expression in *Pseudomonas aeruginosa* remains relatively stable in mature (after 14 h) biofilms [38].

## 5. Conclusions

In conclusion, it could be demonstrated that low concentrations of cadmium, which do not affect bacterial viability, act as stressors that stimulate biofilm formation of *S. epidermidis*. This is of clinical interest, as previous investigations have shown that approximately 50% of all clinical isolated of *S. epidermidisa* produce biofilms and that this biofilm formation does not correlate in all cases with the presence of the *ica*A/D operon. Hence further investigations should clarify if cadmium is one of the stress factors provoking biofilm formation also in strains that do not form biofilms under stress-free conditions. Considering that human exposure to cadmium occurs often by inhalation (occupational exposure or smoking), its biofilm-stimulating effect may be an important factor in the pathogenesis of chronic bacterial airway infections and the resistance of these infections to therapeutic approaches.

## Supplementary Files

Supplementary File 1

## References

[1] Jarup L. (2003). Hazards of heavy metal contamination. Br. Med. Bull..

[2] Zhao H., Xia B., Fan C., Zhao P., Shen S. (2012). Human health risk from soil heavy metal contamination under different land uses near Dabaoshan mine, Southern China. Sci. Total Environ..

[3] Wang B., Du Y. (2013). Cadmium and its neurotoxic effects. Oxid. Med. Cell. Longev..

[4] Wang Q., Zhang J., Zhao B., Xin X., Zhang C., Zhang H. (2014). The influence of long-term fertilization on cadmium (Cd) accumulation in soil and its uptake by crops. Environ. Sci. Pollut. Res. Int..

[5] JECFA Cadmium. Evaluations of the Joint FAO/WHO Expert Committee on Food Additives (JECFA). http://apps.who.int/food-additives-contaminants-jecfa-database/PrintPreview.aspx?chemID=1376.

[6] Perrin C., Briandet R., Jubelin G., Lejeune P., Mandrand-Berthelot M.A., Rodrigue A., Dorel C. (2009). Nickel promotes biofilm formation by *Escherichia coli* K-12 strains that produce curli. Appl. Environ. Microbiol..

[7] Coenye T., Nelis H.J. (2010). *In vitro* and *in vivo* model systems to study microbial biofilm formation. J. Microbiol. Methods.

[8] Fey P.D., Olson M.E. (2010). Current concepts in biofilm formation of *Staphylococcus epidermidis*. Future Microbiol..

[9] Rabah A.B., Oyeleke S.B., Manga S.B., Hassan L.G., Ijah U.J.J. (2010). Microbiological and physico-chemical assessment of soil contaminated with abattoir effluents in sokoto metroplis, Nigeria. Sci. World J..

[10] Ayed L., Chaieb K., Cheref K., Bakhrouf A. (2010). Biodegradation and decolorization of triphenylmethane dyes by *Staphylococcus epidermidis*. Desalination.

[11] Cargill J.S., Upton M. (2009). Low concentrations of vancomycin stimulate biofilm formation in some clinical isolates of *Staphylococcus epidermidis*. J. Clin. Pathol..

[12] Schue M., Fekete A., Ortet P., Brutesco C., Heulin T., Schmitt-Kopplin P., Achouak W., Santaella C. (2011). Modulation of metabolism and switching to biofilm prevail over exopolysaccharide production in the response of *Rhizobium alamii* to cadmium. PLoS One.

[13] Gill S.R., Fouts D.E., Archer G.L., Mongodin E.F., Deboy R.T., Ravel J., Paulsen I.T., Kolonay J.F., Brinkac L., Beanan M. (2005). Insights on evolution of virulence and resistance from the complete genome analysis of an early methicillin-resistant *Staphylococcus aureus* strain and a biofilm-producing methicillin-resistant *Staphylococcus epidermidis* strain. J. Bacteriol..

[14] Mertens A., Ghebermedhin B. (2013). Genetic determinants and biofilm formation of clinical *Staphylococcus epidermidis* isolates from blood cultures and indwelling devises. Eur. J. Microbiol. Immunol..

[15] Melchior M.B., Vaarkamp H., Fink-Gremmels J. (2006). Biofilms: A role in recurrent mastitis infections?. Vet. J..

[16] Wu X., Santos R.R., Fink-Gremmels J. (2014). *Staphylococcus epidermidis* biofilm quantification: Effect of different solvents and dyes. J. Microbiol. Methods.

[17] Cabal B., Cafini F., Esteban-Tejeda L., Alou L., Bartolome J.F., Sevillano D., Lopez-Piriz R., Torrecillas R., Moya J.S. (2012). Inhibitory effect on *in vitro*
*Streptococcus oralis* biofilm of a soda-lime glass containing silver nanoparticles coating on titanium alloy. PLoS One.

[18] Wang X.M., Noble L., Kreiswirth B.N., Eisner W., McClements W., Jansen K.U., Anderson A.S. (2003). Evaluation of a multilocus sequence typing system for *Staphylococcus epidermidis*. J. Med. Microbiol..

[19] Vandecasteele S.J., Peetermans W.E., R Merckx R., Rijnders B.J., Van Eldere J. (2003). Reliability of the ica, aap and atlE genes in the discrimination between invasive, colonizing and contaminant *Staphylococcus epidermidis* isolates in the diagnosis of catheter-related infections. Clin. Microbiol. Infect..

[20] Vandecasteele S.J., Peetermans W.E., Merckx R., Van Eldere J. (2001). Quantification of expression of *Staphylococcus epidermidis* housekeeping genes with taqman quantitative PCR during *in vitro* growth and under different conditions. J. Bacteriol..

[21] Patel J.D., Colton E., Ebert M., Anderson J.M. (2012). Gene expression during *S. epidermidis* biofilm formation on biomaterials. J. Biomed. Mater. Res. A.

[22] Mekni M.A., Bouchami O., Achour W., Ben Hassen A. (2012). Strong biofilm production but not adhesion virulence factors can discriminate between invasive and commensal *Staphylococcus epidermidis* strains. APMIS.

[23] Tormo M.A., Marti M., Valle J., Manna A.C., Cheung A.L., Lasa I., Penades J.R. (2005). SarA is an essential positive regulator of *Staphylococcus epidermidis* biofilm development. J. Bacteriol..

[24] Frebourg N.B., Lefebvre S., Baert S., Lemeland J.F. (2000). PCR-based assay for discrimination between invasive and contaminating *Staphylococcus epidermidis* strains. J. Clin. Microbiol..

[25] Christner M., Heinze C., Busch M., Franke G., Hentschke M., Bayard Duhring S., Buttner H., Kotasinska M., Wischnewski V., Kroll G. (2012). sarA negatively regulates *Staphylococcus epidermidis* biofilm formation by modulating expression of 1 MDa extracellular matrix binding protein and autolysis-dependent release of eDNA. Mol. Microbiol..

[26] Knobloch J.K., Bartscht K., Sabottke A., Rohde H., Feucht H.H., Mack D. (2001). Biofilm formation by *Staphylococcus epidermidis* depends on functional RsbU, an activator of the sigB operon: Differential activation mechanisms due to ethanol and salt stress. J. Bacteriol..

[27] Arciola C.R., Campoccia D., Gamberini S., Donati M.E., Pirini V., Visai L., Speziale P., Montanaro L. (2005). Antibiotic resistance in exopolysaccharide-forming *Staphylococcus epidermidis* clinical isolates from orthopaedic implant infections. Biomaterials.

[28] Shaivastave A., Singh V., Jadon S., Bhadauria S. (2013). Heavy metal tolerance of three different bacteria isolated from industrial effluent. Int. J. Pharm. Bio-Sci..

[29] AbubAkr S., Crupper S.S. (2010). Prevalence of cadmium resistance in *Stahphylococcus sciuri* isolated from the Gray Treefrog, *Hyla chrysoscelis* (Anura: Hylidae). Phyllomedusa.

[30] Teitzel G.M., Parsek M.R. (2003). Heavy metal resistance of biofilm and planktonic *Pseudomonas aeruginosa*. Appl. Environ. Microbiol..

[31] Delumeau O., Dutta S., Brigulla M., Kuhnke G., Hardwick S.W., Volker U., Yudkin M.D., Lewis R.J. (2004). Functional and structural characterization of RsbU, a stress signaling protein phosphatase 2C. J. Biol. Chem..

[32] Williams R.J., Henderson B., Sharp L.J., Nair S.P. (2002). Identification of a fibronectin-binding protein from *Staphylococcus epidermidis*. Infect. Immun..

[33] Gerke C., Kraft A., Sussmuth R., Schweitzer O., Gotz F. (1998). Characterization of the N-acetylglucosaminyltransferase activity involved in the biosynthesis of the *Staphylococcus epidermidis* polysaccharide intercellular adhesin. J. Biol. Chem..

[34] Hu J., Xu T., Zhu T., Lou Q., Wang X., Wu Y., Huang R., Liu J., Liu H., Yu F. (2011). Monoclonal antibodies against accumulation-associated protein affect EPS biosynthesis and enhance bacterial accumulation of *Staphylococcus epidermidis*. PLoS One.

[35] Dai L., Yang L., Parsons C., Findlay V.J., Molin S., Qin Z. (2012). *Staphylococcus epidermidis* recovered from indwelling catheters exhibit enhanced biofilm dispersal and “self-renewal” through downregulation of agr. BMC Microbiol..

[36] Dieppois G., Ducret V., Caille O., Perron K. (2012). The transcriptional regulator CzcR modulates antibiotic resistance and quorum sensing in *Pseudomonas aeruginosa*. PLoS One.

[37] Pintens V., Massonet C., Merckx R., Vandecasteele S., Peetermans W.E., Knobloch J.K., Van Eldere J. (2008). The role of sigmaB in persistence of *Staphylococcus epidermidis* foreign body infection. Microbiology.

[38] Waite R.D., Papakonstantinopoulou A., Littler E., Curtis M.A. (2005). Transcriptome analysis of Pseudomonas aeruginosa growth: Comparison of gene expression in planktonic cultures and developing and mature biofilms. J. Bacteriol..

