# Supplementary material for: Cadmium Modulates Biofilm Formation by Staphylococcus epidermidis
Source: Int J Environ Res Public Health. 2015 Mar 4;12(3):2878–94. doi: 10.3390/ijerph120302878 (PMC4377938; doi:10.3390/ijerph120302878)
Supplement: Supplementary File 1 [file ijerph-12-02878-s001.pdf]

Cadmium Modulates Biofilm Formation by *Staphylococcus epidermidis*

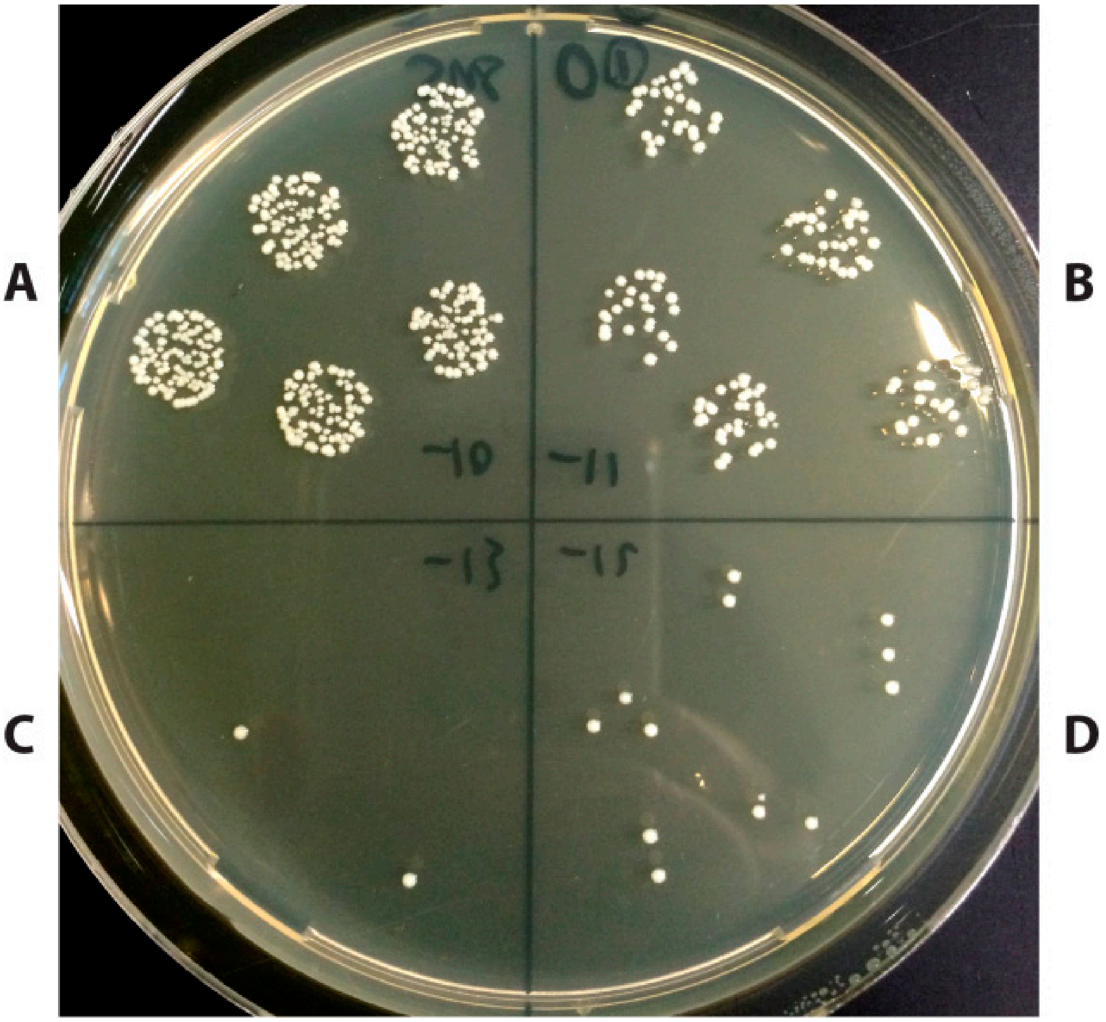

Figure S1. Example of a drop plate assay. Five 10 μL drops of 10^{10}, 10^{11}, 10^{12} and 10^{13} times diluted bacterial suspension were dropped on areas **A**, **B**, **C** and **D**, respectively. Bacterial colonies from each drop can be counted after culture the plate 24 h at 37 °C.

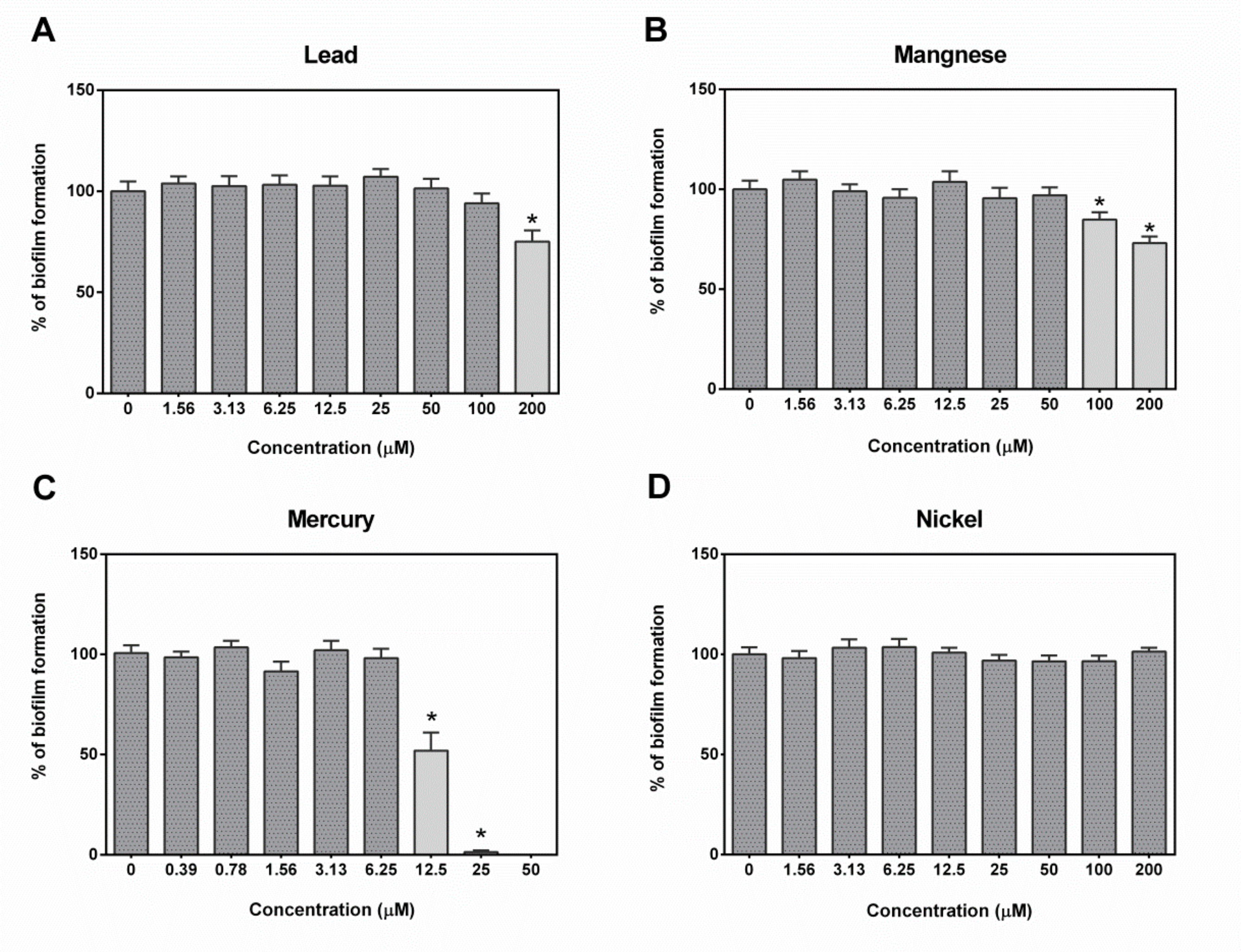

Figure S2. Mean percentage (± SEM) of *S. epidermidis* (ATCC35984) biofilm formation after exposure to 1.56–200 μM lead (**A**), manganese (**B**) or 0.39–50 mercury (**C**), or nickel (**D**). * indicates significant ($p < 0.01$) difference between treatments and control (0 μM).

© 2015 by the authors; licensee MDPI, Basel, Switzerland. This article is an open access article distributed under the terms and conditions of the Creative Commons Attribution license (http://creativecommons.org/licenses/by/4.0/).
